# Tracheal Carinal Reconstruction and Bronchovasculoplasty in Central Type Bronchogenic Carcinoma

**DOI:** 10.3779/j.issn.1009-3419.2010.04.16

**Published:** 2010-04-20

**Authors:** Deruo LIU, Yongqing GUO, Bin SHI, Yanchu TIAN, Zhiyi SONG, Qianli MA, Zhenrong ZHANG, Bingsheng GE

**Affiliations:** Department of Thoracic Surgery, China-Japan Friendship Hospital, Beijing, China

**Keywords:** Lung cancer surgery, Bronchial arteries, Statistics, Survival analysis, Lymph nodes, Lung neoplasms

## Abstract

**Background and objective:**

Because radical resection for lung cancer invading the initial borderline of different lobes and carina is difficult, we tried to analyse the variables of successful tracheal carinoplasty and bronchovasculoplasty to discover a proper approach for appropriate early and long term results.

**Methods:**

Of 1 399 lung resections for primary lung cancer performed in our hospital from April 1985 to December 2006, 133 underwent bronchoplastic surgeries, including 15 carinoplasty cases and 118 sleeve lobectomy (SL) cases, and 118 pneumoectomy (PN) cases were compared at the same time.

**Results:**

Complications occurred in 18 cases, with no operative related mortality. For all patients, the 1 year, 3 year, and 5 year survival rates were 79.8%, 56.7% and 31.2%, respectively. The 5 year survival rate by cancer stage was 69.2% for Ⅰb, 40.6% for Ⅱb, 19.6% for Ⅲa, and 16.6% for Ⅲa (N2).

## Introduction

Sleeve lobectomy and carinal resection are widely accepted as beneficial alternatives to pneumonectomy. They offer maximal preservation of lung function, allowing radical operations in patients who would not tolerate pneumonectomy^[1, 2]^. They are used for the treatment of benign lung tumors, low grade carcinoma, tuberculosis, metastatic tumors and cancer^[3]^. We report the techniques employed and patient survival for all bronchoplasty procedures for lung resection performed in our center over 21 years period.

## Patients and methods

### Patient data

Our Institutional Review Board approved this study on February 2008 and individual consent was waived. During April 1985 to December 2006, 1 399 lung resections for primary lung cancer were performed in the Department of Thoracic Surgery, China-Japan Friendship Hospital. Of these, 133 cases underwent bronchoplastic surgery, including 15 partial resections of the bifurcation (carinoplasty) and 118 sleeve lobectomy cases. Patients were mostly male (10 132 female), aged 19-73 years. The pathological classification and p-TNM stages are shown in Tab 1. The reason why we choose some T2 and T3 central type lung cancer patients for sleeve lobectomy is that the tumor invade the initial borderline of upper lobe, lower lobe, and bronchus intermedius. Routine procedure of lobectomy can not ensure the stump negative, but sleeve lobectomy can remove the whole bronchus tumor, so bronchoanastoma is free of tumor cells. Because of this reason, this type of operation is applied, no matter the distance between the tumor and carina is more than (T2) or less than (T3) 2 cm.

**1:** Histological diagnosis of patients, p-TNM stage and types of bronchoplastic procedures (*n*=133)

Characterastics		Carinal resection	Bronchoplasty
Histological classification	Squamous cancer	13	87
	Adenocarcinoma	2	17
	Small cell lung cancer	0	7
	Low grade carcinoma	0	3
	Adenosquamous carcinoma	0	4
Stage	Ⅰb T2N0M0	0	22
	Ⅱb T2N1M0	0	31
	T3N0M0	0	8
	Ⅱb T2N2M0	0	13
	Ⅲa T3N0M0	0	30
	T3N1M0	11	7
	T3N2M0	4	7

### Selection of patients

Obvious symptoms were always found if the tumor invaded carina or proximal main bronchus. Serious complications might occur in a short time and life was threatened, so emergent treatment should be taken. Resection and reconstruction of the airway were effective methods.

Rationale for operating: Serious obstruction and dyspnea were often found before operation and always accompanied with obstructive pneumonia, fever and cough. In such cases, conservative treatment accomplished little and survival was short.

Cases selected for operation: Inclusion criteria included the following: most (126) cases are non small cell lung cancer (NSCLC), 7 cases are small cell lung cancer (SCLC)(after chemotherapy). The clinical and p-TNM stage is T2/T3, N0/N1/ N2, M0. The reason why some T2 and T3 patients were chosen for sleeve lobectomy is that the tumor locate in the canal orifice of upper lobe bronchus, bronchus intermedius or lower lobe bronchus, no matter the distance between the tumor and carina is more or less than 2 cm, routine procedure of lobectomy can not ensure the stump negative, but sleeve lobectomy can remove the whole tumor and ensure the bronchus stoma is free of tumor cells. Surgical indication of N2 is unexpected N2 (found after operation) and proven N2 (proven before operation, operation is carried out after two times of neochemotherapy), group of bulky metastatic mediastinal lymph nodes is not included. The main blood vessels are evaluated "not invaded" through enhanced CT scan of the chest preoperatively. Surgeons observed the sphere and length of tumor by fibrobronchoscope, confirming the possibility of airway reconstruction after resection.

Generally speaking, pneumonectomy was necessary in cases with centrally located lesions, as the tumor extended directly into the mediastinum, canal orifice of bronchus or mucous membrane nearby (mostly the bilateral upper lobe). When patients' lung function is too low to bear the pneumonectomy, and the extensive tumor invasion makes it difficult for routine lobectomy to obtain sufficient bronchial margins for negative stump, the special operation, sleeve lobectomy, is an appropriate choice for the goal of radical resection with minimum lung function loss.

### Preoperative evaluation

Routine examination included bronchoscopy, chest radiography and CT (computed tomography) scan of the chest, upper abdomen and head, and cardiac and pulmonary function. The 15 carinal resection cases were symptomatic with fever, expectoration with blood, or dyspnea. Exceptional conditions included esophagobronchial fistula (1 case), compromised pulmonary function (2 cases), and ECG showing ST-T changes (1 case).

### Anesthesia techniques

Carinal resection: While most cases were uneventful applied standard anesthesia techniques, single-lumen endotracheal tube was used in 5 cases, single-lumen endotracheal tube together with high frequency oscillation in 6 cases, and double-lumen endotracheal tube in 4 cases.

Sleeve lobectomy: Double-lumen endotracheal tube was used in 61 cases, and single-lumen endotracheal tube was used in 19 cases. Briefly, most patients were assigned to undergo either right upper sleeve lobectomy (60 cases) or left upper sleeve lobectomy (40 cases). Much fewer were assigned to left lower sleeve lobectomy (8 cases) or right pneumonectomy with carinoplasty (7 cases). Remaining procedures were performed 5 or fewer times during the study period.

### Operative techniques

Sleeve lobectomy: Details of operative techniques are shown in Fig 1. Depending upon the section to be operated upon, the surgeon selects the area to be excised, cutting above the area following the curve of the bronchia above: using acute angles above and below for sleeve lobectomy of right upper lobe (A), and sleeve lobectomy of left upper lobe (B), steeper angles for sleeve bilobectomy of right middle and lower lobes (C), right sleeve pneumonectomy (E) and sleeve bilobectomy of right upper and middle lobes with lateral wall resection of inferior trachea, carinal carinoplasty (F). Areas allowing for greater preservation of area below the lesion, such as sleeve lobectomy of left lower lobe (D), require suturing sections together for right pneumonectomy with bronchoplasty (G) and left pneumonectomy with bronchoplasty (H). Bronchus wedge resection may induce angulated or constrictive stoma, the length of resection is limited, and the resected edges can be positive. In such cases we use full sleeve lobectomy to resect the bronchus circularly. In situations with positive resection edge and compromised lung function, an extended resection should be applied. The anastomosis of the bronchi is usually covered by a pedicled intercostal muscle flap to prevent complications of the anastomosis. Bronchoplasty techniques: 1) After disposal of the pulmonary vessel, the length of resection is carefully schemed in a clear field, to make sure the two ends can be anastomosed safely. 2) For giant tumors, pneumonectomy and carinal plasty might be applied. The field of vision is usually not very clear, so the lobe in which the tumor is located is resected first, and then end-to-end anastomosis is performed after chipping.

**1:**
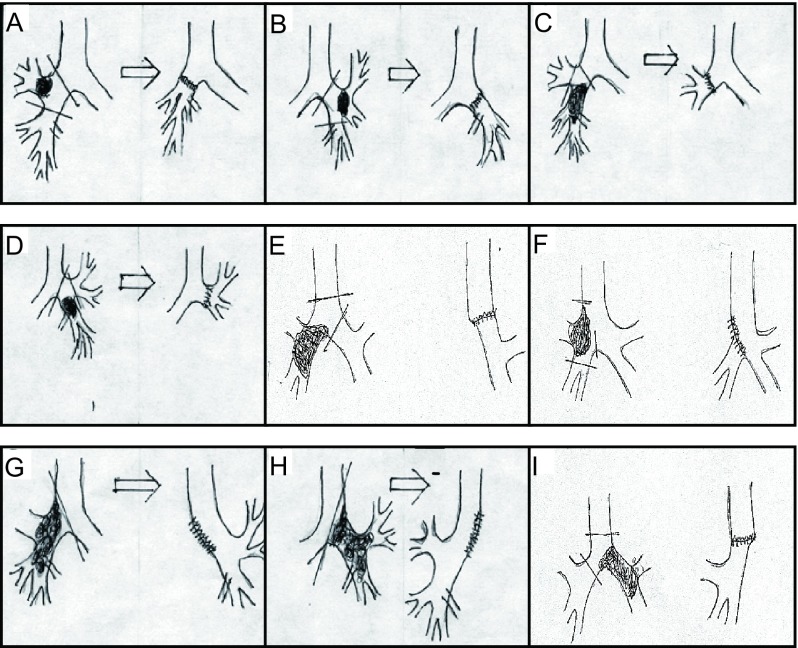
Sleeve lobectomy procedures used for lung cancer

Procedure for blood vessel: Details are shown in Fig 2. Different operation types are as follows: right pulmonary artery plasty (RPAP, *n*=1), left pulmonary artery plasty (LPAP, *n*=5), RPAP of lateral wall (*n*=7), LPAP of lateral wall (*n*=4), left upper bronchus sleeve resection with resection of lingual segment artery (*n*=1), right middle lower bronchus sleeve resection with resection of posterior ascending artery (*n*=5). In all, 23 PA plasty operations were performed. Specific procedures applied during operation are shown in Fig 2. And three key points must be remembered: 1) It is safe to dissect areas not invaded by the tumor, where the tissue appears normal. At times dissection is done through the inside of the cardinal sac. 2) The circumference of the blood vessel to be ligated later must be dissected with sharp or blunt methods. The root of the proximal pulmonary artery is ligated, and the distal bifurcation ligated respectively to avoid ablation. 3) If the superior vena cava is partly invaded, three methods can be used: First, for minimal involvement, a side-biting clamp can be applied and the cava simply sutured or patched if the lumen is compromised. Second, insert a silica tube into the lateral aperture so that the distal end exceeds the tumor. Finally, if the lateral aperture is in the right atrium, construct an internal shunt after cross-clamping. Once the procedure has been performed, excise the tumor, and repair the defect.

**2:**
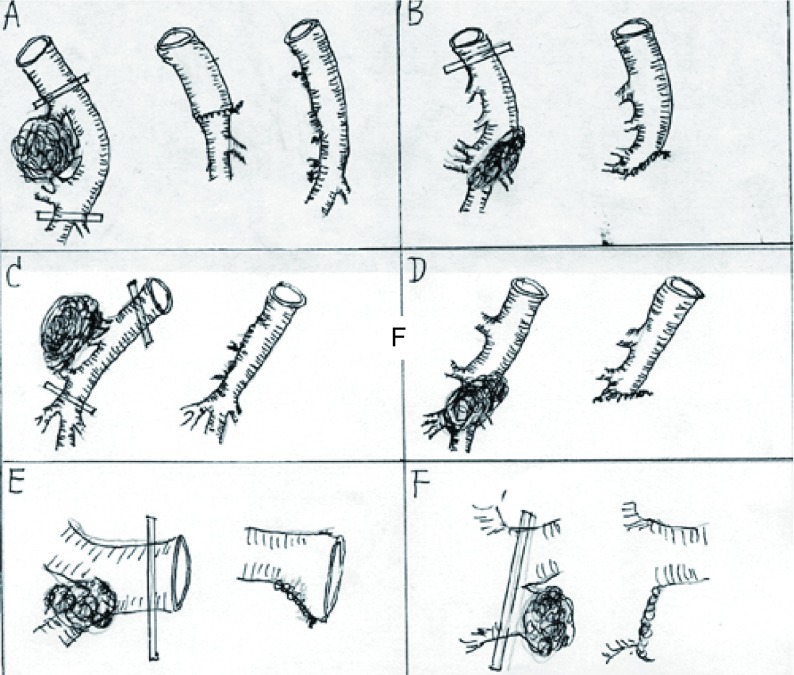
Types of PA plasty

Clearance of lymph nodes: It is suggested that lymph nodes of the hilum should be cleared before bronchus anastomosis, the advantages are as follows: 1) resection can be completed, especially for the subcarinal tissue, 2) the operative field is clear, 3) anastomosis is made more convenient. After clearance, the mediastinal lymph nodes and fatty tissue are resected. Metal clip markers are placed for postoperative radiotherapy if doubtful tissues are left.

All patients in this group received post operative chemotherapy, and neochemotherapy is applied for proven N2 patients. The indication of local radiotherapy postoperatively is all N2 patients and those with a positive incision edge.

## Results

For all 118 cases undergoing sleeve lobectomy, atelectasis occurred in 13 cases, arrhythmia occurred in 4, stomal fistula occurred in 1 case and 1 patient died in 30-day after operatin. At the same time, 118 cases, underwent pneumoectomy in the same period, are compared with sleeve lobectomy, the results are as follows: 30-day postoperative mortality of the pneumonectomy (PN) group and sleeve lobectomy (SL) group were 5.9% and 0.8%, respectively (*P*=0.031). There were no differences in postoperative complications (SL, 16.1% *vs* PN, 26.3%, *P*=0.056) and local recurrences (SL, 6.8% *vs* PN, 7.6%, *P*=0.080). The overall 5-year survivals for the SL group were 45%, whereas those for the PN group were 24% (*P*=0.005 7), (Tab 2). These data suggest that sleeve lobectomy should be performed instead of pneumonectomy in patients with nonsmall cell lung cancer regardless of their nodal status whenever complete resection can be achieved because this is a lung-saving procedure with lower postoperative risks and is as curative as pneumonectomy.

**2:** Compare between sleeve lobectomy and pneumoectomy (*n*=118)

	Sleeve lobectomy	Pneumoectomy	*P*
30-day postoperative mortality	0.8% (1/118)	5.9% (7/118)	0.031
Postoperative complication	16.1% (19/118)	26.3% (31/118)	0.056
Local recurrences	6.8% (8/118)	7.6% (9/118)	0.080
5-year survivals	45%	24%	0.057

The 5 year survival rate by TNM stage for patients undergoing tracheal carinal reconstruction and bronchovasculoplasty was 69.2% for Ⅱb, 40.6% for Ⅱb, 19.6% for Ⅲa, and 16.6% for Ⅲa (N2) respectively, (*P* < 0.05). Survival curves for 118 cases with sleeve lobectomy and 15 cases with carinoplasty according TNM stage are shown in Fig 3 and Fig 4.

**3:**
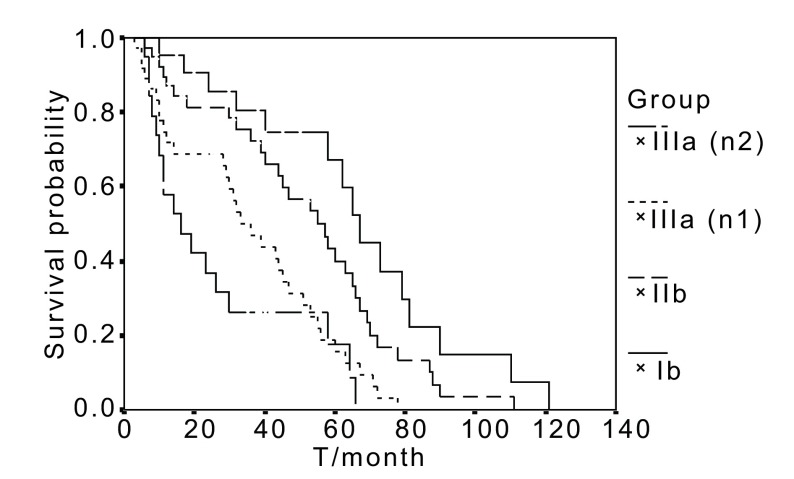
Survival curves for 118 cases with sleeve lobectomy according to TNM stage There are significant differences between 4 groups (*P* < 0.05).

**4:**
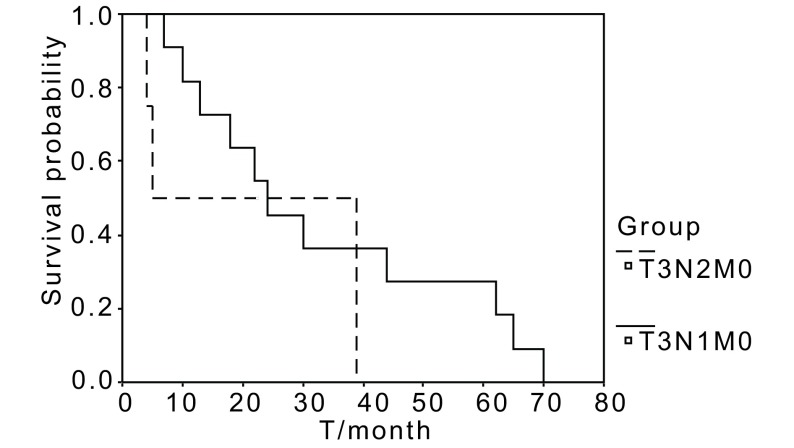
Survival curves for 15 cases with carinoplasty according to TNM stage There are significant differences between 4 groups (*P* > 0.05).

Follow up: 8 cases (SL group) had local recurrence, and stomal fistula occurred in 1 case (SL group). Of 15 cases, who underwent carinoplasty, 4 had difficulty discharging the excretion of the airway; all recovered after aspiration via bronchoscope.

## Comment

Sleeve lobectomy and carinal resection are widely accepted as beneficial alternatives to pneumonectomy, and patient life quality is improved while maximal lung function is preserved. Many investigators have reported that lobectomy with bronchoplasty has similar or fewer postoperative complications, similar or better long-term survival, and superior residual pulmonary function compared to pneumonectomy^[4-6]^. Thus, whenever possible, sleeve lobectomy is recommended and in any case, a macroscopically sufficient margin of safety is required^[7]^.

The overall 5-year survival rate of sleeve resection has been reported at 30%-40%. Some have demonstrated that survival rate depends more on disease stage than resection technique. Long-term survival is particularly influenced by the extent of metastasis in the hilar (N1) and mediastinal nodes (N2); most such patients die of distant metastases^[8, 9]^.

It is reported that 5 and 10-year survival rates for N0 cases are 72.4% and 59.4%, 22% and 14.4% for N2 cases. They were similar to our findings. Our postoperative mortality was 0.8% compared with the 2%-6.2% reported by other authors, and postoperative complications were fewer^[6]^. In our group, one stomal fistula was cured conservatively. Patients with atelectasis and difficulty in expectoration were all treated with aspiration through bronchoscope, trachea incision was avoided.

Our low complication rate after operation was related to the measures we took. In particular, the frozen section should be confirmed if a sample was not acquired preoperatively. To avoid excessive lung resection, we carefully examined the invasive sphere of the tumor and mediastinal lymph nodes in every case, evaluating whether the patient absolutely needed radical bronchoand pulmonary resection or anastomosis.

In conclusion, bronchoplastic procedures require exact selection of cases, preoperative evaluation, high surgical skill, and intensive postoperative care. Operative morbidity is very low, and mortality figures compare favorably with those of lung resection for malignant and benign conditions. Where indications exist, they should be freely performed.

## Acknowledgement

This study was supported by grants from the Science and Research Department, belongs to China-Japan Friendship Hospital directly affiliated to Chinese Ministry of Health.
